# Evaluating Partial Middle Ear Filling for the Diagnosis of Cholesteatoma Using Machine Learning: A Radiological Approach

**DOI:** 10.7759/cureus.83503

**Published:** 2025-05-05

**Authors:** Naouar Ouattassi, Abdelhay Zoizou, Mustapha Maaroufi, El Mehdi Labiyad, Taha Benatiya Andaloussi, Najib Benmansour, Mohammed Ridal, Arsalane Zarghili, Mohamed Nouredine El Amine El Alami

**Affiliations:** 1 Biomedical and Translational Research Laboratory, Faculty of Medicine, Pharmacy and Dentistry, University Sidi Mohamed Ben Abdellah, Fez, MAR; 2 Otolaryngology - Head and Neck Surgery Department, Hassan II University Hospital, Fez, MAR; 3 Laboratory of Science, Engineering and Management, Higher School of Technology, University Sidi Mohammed Ben Abdellah, Fez, MAR; 4 Radiology Department, Hassan II University Hospital, University Sidi Mohamed Ben Abdellah, Fez, MAR; 5 Otolaryngology - Head and Neck Surgery Department, Hassan II University Hospital, University Sidi Mohamed Ben Abdellah, Fez, MAR; 6 Computer Science Department, Intelligent Systems and Applications Laboratory, Faculty of Science and Techniques, University Sidi Mohamed Ben Abdellah, Fez, MAR

**Keywords:** artificial intelligence, computed-tomography, machine learning, middle ear cholesteatoma, temporal bone

## Abstract

Objectives. To evaluate the diagnostic accuracy of middle ear partial filling on temporal bone computed tomography (CT) scan for middle ear cholesteatoma identification using supervised machine learning models.

Methods. We conducted an observational case-control study that retrospectively analyzed temporal bone CT scans from 212 patients from a single tertiary healthcare institution using supervised machine learning models, including k-Nearest Neighbors (kNN), Neural Networks, Logistic Regression, Support Vector Machine (SVM), and Random Forest. The study assessed the diagnostic value of partial middle ear filling for cholesteatoma. Limitations such as dataset imbalance and data complexity were addressed.

Results. In internal validation, kNN and Neural Networks achieved the highest performance (area under the receiver operating characteristic curve [AUC]: 1.000, classification accuracy [CA]: 99.6-99.7%, F1: 0.996-0.997), followed by Logistic Regression (AUC: 0.998, CA: 98.3%, F1: 0.983) and SVM (AUC: 0.997, CA: 97.5%, F1: 0.975). Random Forest performed the weakest (AUC: 0.980, CA: 92.0%, F1: 0.919). External validation (125 cases) revealed Neural Networks’ superior generalizability (four errors), outperforming Logistic Regression (five), SVM (seven), Random Forest (28), and kNN (45). kNN demonstrated notably lower generalizability, suggesting limited robustness for unseen data.

Discussion. The study highlights the effectiveness of machine learning in diagnosing cholesteatoma. Addressing data imbalance and variability in CT scans was crucial for model performance. Further research is needed to refine these models and explore their integration into clinical practice.

## Introduction

Middle ear cholesteatoma is an inflammatory disorder characterized by the presence of a matrix in the middle ear cavities, which secretes inflammatory and osteolytic mediators in response to secondary infections, often involving biofilm formation. This pathological process disrupts the migration of the keratinizing epithelium of the tympanic membrane and inner part of the external auditory canal, leading to the accumulation of keratin debris and subsequent bone destruction, with the potential for severe complications [1]. In contrast, suppurative chronic otitis media (SCOM) typically presents with otorrhea and/or hearing loss and lacks the characteristic osteolytic progression of cholesteatoma, although it may also result in significant complications in some cases.

The diagnosis of middle ear cholesteatoma is primarily clinical, identified through otoscopy, which reveals keratin in the tympanic cavity and is often associated with retraction pockets [2]. Temporal bone CT scans are critical for evaluating the extent of bone destruction and the involvement of the middle ear's posterior cavities, assisting in surgical planning [3]. Additionally, CT scans are useful when otoscopic findings are inconclusive or when anatomical limitations obscure the tympanic membrane [3]. While CT scans have high sensitivity in detecting middle ear pathology, their specificity remains moderate, and accurate interpretation requires specialized training and experience [4].

Artificial intelligence (AI) technology has significantly influenced medicine by improving diagnostic accuracy and enhancing clinical decision-making, particularly as medical data volumes increase [5]. However, in otology, few studies have applied machine learning (ML) or deep learning models to diagnose middle ear cholesteatoma using CT scan image analysis [6-13].

In cases of partial middle ear filling, conventional CT interpretation may lack specificity and rely heavily on the radiologist's experience. Given the diagnostic ambiguity and potential for variability in human interpretation, ML models may offer a standardized and reproducible approach to aid in the detection of middle ear cholesteatoma. We hypothesize that supervised ML models can accurately identify cholesteatoma based on partial middle ear filling patterns observed in temporal bone CT scans.

The primary objective of this study was to evaluate the diagnostic accuracy of five supervised ML models (k-Nearest Neighbors [kNN], Neural Network, Logistic Regression, Support Vector Machine [SVM], and Random Forest) in distinguishing cholesteatoma from SCOM, and to compare their performance on both internal and external validation datasets in order to assess their potential utility in clinical decision-making.

## Materials and methods

Study design

We conducted a retrospective, observational case-control study to analyze pre-existing medical imaging data, comparing partial middle ear filling in patients with cholesteatoma to those with SCOM. The study protocol was approved by our Institutional Review Board, and an informed consent waiver was granted.

Population and sample

The dataset for this study included a total of 212 patients divided into two groups: 102 patients operated on for middle ear cholesteatoma and 110 patients operated on for SCOM. These patients provided a total of 3,398 temporal bone CT scan images, including 561 images from patients with cholesteatoma and 2,837 images from patients with SCOM.

The dataset used in our study comprised native temporal bone CT scans with a slice thickness of 1 millimeter. These scans were retrieved as Digital Imaging and Communication in Medicine (DICOM) files from the hospital's Picture Archiving and Communication System (PACS) and were anonymized prior to analysis. For the purpose of this study, we specifically utilized axial views, which were obtained using a 32-channel multidetector BrightSpeed CT scanner (GE Medical Systems, France).

Data collection and preparation

*Annotation* 

Key slices from the temporal bone CT scan series were manually selected and annotated by two specialists, an otolaryngologist and a radiologist with five- and ten-year experience, respectively. Image classification was based on established CT features commonly associated with middle ear cholesteatoma, including scutum erosion, widening of the aditus, and soft tissue filling of the epitympanum or mastoid. Both specialists relied on these standard radiologic signs during manual annotation. Although a formal checklist was not used, image labeling was consistent with clinical criteria found in the otologic imaging literature [2]. Also, the two experts independently categorized each image as either “cholesteatoma” or “SCOM.” 

During the annotation phase, both experts were blinded to surgical outcomes, and no access to clinical data or operative reports was allowed. Each expert’s labeling was then cross-referenced with the patient’s surgical findings, which served as the definitive diagnosis. In case of disagreement between the expert’s classification and the surgical report, the latter was used to assign the final diagnostic label. No consensus process or mutual comparison between experts was performed in order to preserve the objectivity of the annotation and avoid interpretative bias. Pertinent images were ultimately saved in JPEG format. 

Preprocessing

Images were normalized to standardize pixel value scales. Artifacts were removed. Regions of interest (ROI) in the middle ear were segmented and isolated for further processing. 

The segmentation of the middle ear region was performed manually by selecting key axial slices that displayed partial middle ear filling. This was done visually by trained specialists using anatomical landmarks such as the epitympanum, mastoid air cells, and ossicular chain. 

Dataset Construction

The images were divided into three distinct sets, 70% for training, 20% for testing, and 10% for validation. Training and testing datasets are used to build the model, while the validation set is used to evaluate the performance of the final model.

Addressing Data Imbalance

In our study, addressing data imbalance was crucial since the cholesteatoma group comprised only 16% of the 3,379 total images. To mitigate this, we applied preprocessing and augmentation techniques. Right ear images were mirrored to resemble left ear images, reducing variability and simplifying the classification task. Oversampling was used to balance the dataset, resulting in 3,412 cholesteatoma images and 2,858 SCOM images. Traditional augmentation methods, such as rotation and zooming, were avoided as they introduced distortions that could compromise diagnostic accuracy. 

While advanced synthetic augmentation methods such as Generative Adversarial Networks (GANs) are emerging in medical imaging, their application to middle ear CT scans remains poorly validated and carries a significant risk of generating anatomically implausible images. Given the absence of domain-specific GAN frameworks for temporal bone imaging, we opted for more conservative augmentation methods that preserve clinical realism. SMOTE-based approaches were also tested but did not yield sufficient quality for inclusion. Thus, oversampling and anatomical mirroring, while preserving image realism, were prioritized.

Image processing 

In our study, we used the Inception V3 model, a pre-trained 42-layer convolutional neural network (CNN), to perform "image embedding extraction" and determine image vectors. Inception V3 acts as a feature extractor, condensing image information into a vector of 2048 parameters that summarize key characteristics of each image. This dimensionality reduction facilitates efficient comparison of images. The classification process then involved comparing these embeddings to identify similar images.

ML models

Five ML models were tested: Logistic Regression, kNN, SVM, Random Forest, and Neural Network.

Logistic Regression

Logistic regression is valued for its efficiency and interpretability in binary classification tasks. However, it may be less effective in cases where the relationship between features and outcomes is non-linear [14,15]. Hyperparameter tuning was conducted using a grid search over the regularization strength parameter (C), ranging from 0.01 to 10 on a logarithmic scale. The best value of C = 1.0 was selected based on the highest mean F1 score during 10-fold cross-validation. Least Absolute Shrinkage and Selection Operator (Lasso) (L1) regularization was chosen to enforce sparsity and improve generalization.

k-Nearest Neighbors (kNN)

The kNN algorithm is a nonparametric method used for both classification and regression tasks. It classifies a new observation by identifying the "k" closest data points in the feature space. For classification, it assigns the most frequent class among the neighbors, and for regression, it averages the target values. The value of "k" is a hyperparameter, typically selected through cross-validation, with common values being 3, 5, 7, or 9. The algorithm's performance is influenced by both the choice of "k" and the distance metric (e.g., Euclidean or Manhattan) [14,15]. The value of k and the distance metric were selected via grid search optimization, evaluating k =3, 5, 7, and 9 across several metrics including Euclidean, Manhattan, and Chebyshev distances. The combination of k = 5 with Euclidean distance yielded the highest mean F1 score during 10-fold cross-validation on the training dataset and was therefore used in the final model. 

Support Vector Machine (SVM)

SVM is a robust supervised learning algorithm used for both classification and regression tasks, though it is primarily employed for classification. The algorithm’s performance is sensitive to the selection of hyperparameters, particularly the regularization parameter (C) and the choice of kernel [14,15]. We performed a grid search over the regularization parameter (C = 0.1, 1, 10) and kernel types (linear, polynomial, RBF [Radial Basis Function]). The optimal configuration was found to be C = 1 and an RBF kernel, which yielded the best F1 score during 10-fold cross-validation. The epsilon-insensitive loss (ε = 0.10) was selected to tolerate small prediction errors.

Random Forest

Random Forest is an ensemble learning technique that builds multiple decision trees during training and combines their outputs to improve prediction accuracy. It is versatile, applying to both classification and regression tasks [14,15]. We explored variations in the number of estimators (trees) (10, 50, 100) and maximum tree depth (3, 5, 10). The best performance was achieved with 10 trees and a maximum depth of 3, selected using 10-fold cross-validation and F1 score as the selection criterion. This configuration minimized overfitting while retaining good classification performance.

Neural Network

The neural network model employed in this study is a Multi-Layer Perceptron (MLP). Its architecture was optimized by testing various hidden layer sizes (50, 100, 150 neurons) and activation functions (ReLU [Rectified Linear Unit], tanh). The final model used one hidden layer of 100 neurons with ReLU activation, trained using the Adam (Adaptive Moment Estimation) optimizer with default learning rate and batch size. This configuration achieved the best F1 score under 10-fold cross-validation.

All hyperparameters were selected via grid search optimization using 10-fold cross-validation and F1 score as the primary performance metric to reduce overfitting and ensure optimal and reproducible results.

Model evaluation

The performance of each classification model was evaluated using various metrics, including classification accuracy, recall (sensitivity), precision, F1 score, AUC (area under the receiver operating characteristic curve), and the confusion matrix. The F1 score, the harmonic mean of precision and recall, is especially valuable in scenarios with imbalanced class distributions or where the cost of false positives and false negatives differs. This metric captures the tradeoff between precision and recall, making it crucial when accuracy alone may be misleading. Consequently, the F1 score, alongside accuracy, was used as a primary metric to assess the performance of our models [14,15].

External validation

Each trained model was tested on an independent dataset of 125 temporal bone CT scan images to evaluate its generalizability and robustness.

## Results

Table 1 summarizes the performance metrics of our models after undergoing 10-fold cross-validation. 

**Table 1:** The performance metrics of our models after undergoing tenfold cross-validation AUC: area under the receiver operating characteristic curve; CA: classification accuracy; kNN: k-Nearest Neighbors; SVM: Support Vector Machine.

Model	AUC	CA	F1	Precision	Recall
kNN	1.000	0.996	0.996	0.997	0.996
Neural Network	1.000	0.997	0.997	0.997	0.997
Logistic Regression	0.998	0.983	0.983	0.983	0.983
SVM	0.997	0.975	0.975	0.975	0.975
Random Forest	0.980	0.920	0.919	0.923	0.920

Based on the F1 score, the kNN and Neural Network models demonstrate the best performance, suggesting their potential utility in applications where high accuracy in both positive and negative class predictions is paramount. Table 2 and Table 3 display the confusion matrix of both classifiers.

**Table 2:** Confusion matrix of the kNN model SCOM: suppurative chronic otitis media; kNN: k-Nearest Neighbors.

Actual/Predicted	Cholesteatoma	SCOM	Total (Σ)
Cholesteatoma	3,411	0	3,411
SCOM	22	2,835	2,857
Total (Σ)	3,433	2,835	6,268

**Table 3:** Confusion matrix of the Neural Network model SCOM: suppurative chronic otitis media.

Actual/Predicted	Cholesteatoma	SCOM	Total (Σ)
Cholesteatoma	3,408	3	3,411
SCOM	15	2,842	2,857
Total (Σ)	3,423	2,845	6,268

Logistic Regression also shows commendable performance and could be considered for similar tasks. SVM, while effective, falls short of the top performers in balancing false positives and false negatives. Table 4 and Table 5 present the confusion matrices for both classifiers. 

**Table 4:** Confusion matrix of the Logistic Regression model SCOM: suppurative chronic otitis media.

Actual/Predicted	Cholesteatoma	SCOM	Total (Σ)
Cholesteatoma	3,378	33	3,411
SCOM	73	2,784	2,857
Total (Σ)	3,423	2,845	6,268

**Table 5:** Confusion matrix of the Support Vector Machine model SCOM: suppurative chronic otitis media.

Actual/Predicted	Cholesteatoma	SCOM	Total (Σ)
Cholesteatoma	3,337	74	3,411
SCOM	82	2,775	2,857
Total (Σ)	3,419	2,849	6,268

Whereas, Random Forest, despite being a robust classifier in many scenarios, shows relative weaknesses here, possibly requiring adjustments or tuning to improve its F1 score. Table 6 represents the confusion matrix of this classifier.

**Table 6:** Confusion matrix of the Random Forest model SCOM: suppurative chronic otitis media.

Actual/Predicted	Cholesteatoma	SCOM	Total (Σ)
Cholesteatoma	3,305	106	3,411
SCOM	397	2,460	2,857
Total (Σ)	3,702	2,566	6,268

**Figure 1:**
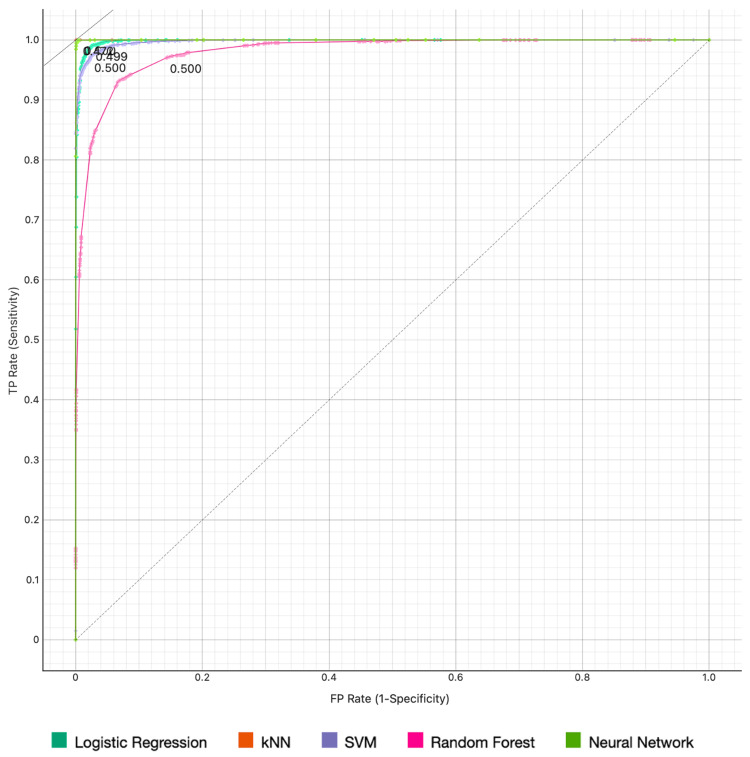
ROC curve for middle ear cholesteatoma Target probability is 54.0%. ROC: receiver operating characteristic; kNN: k-Nearest Neighbors; SVM: Support Vector Machine.

**Figure 2:**
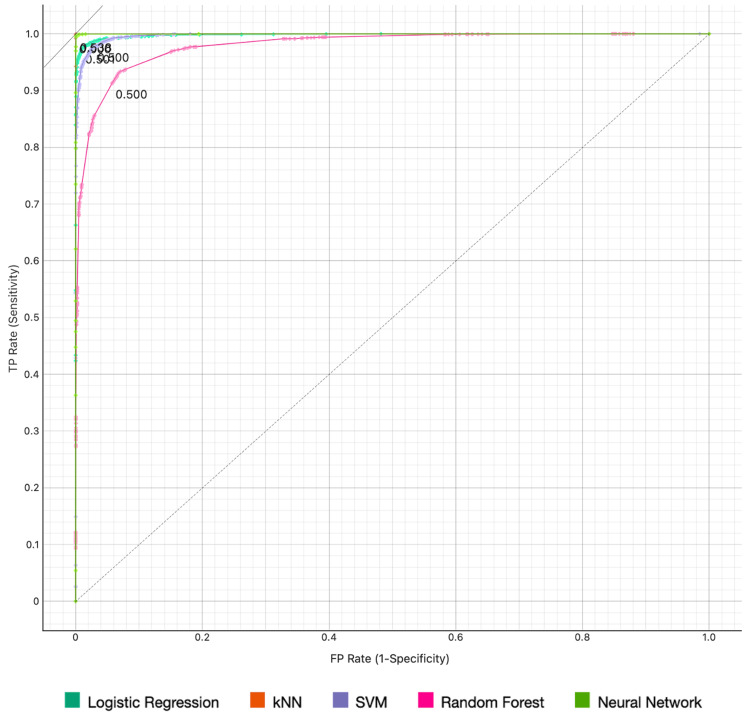
The ROC curve for middle ear suppurative chronic otitis media Target probability is 46.0%. ROC: receiver operating characteristic; kNN: k-Nearest Neighbors; SVM: Support Vector Machine.

Figures 1, 2 display the AUC performance of our models regarding the diagnosis of middle ear cholesteatoma and SCOM, respectively.

**Figure 3:**
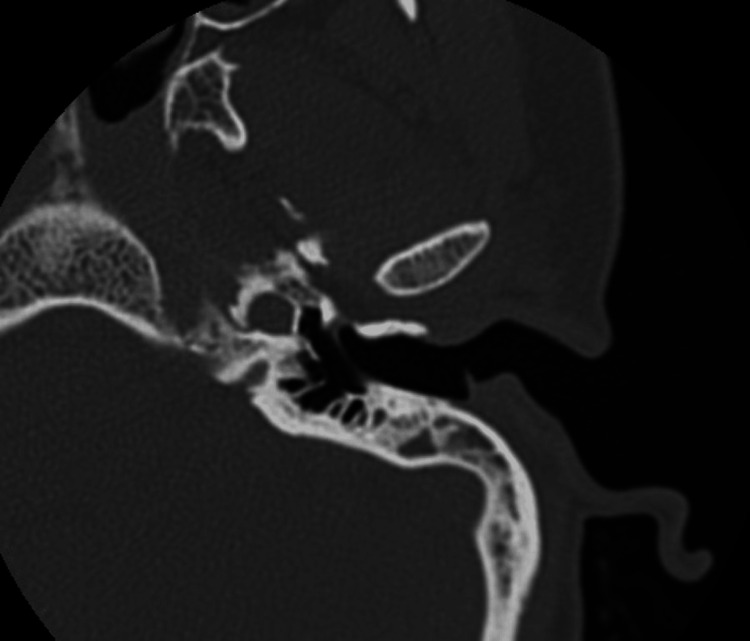
Sample error image of a misclassified SCOM CT scan image using the kNN model SCOM: suppurative chronic otitis media; CT: computed tomography; KNN: k-Nearest Neighbors.

**Figure 4:**
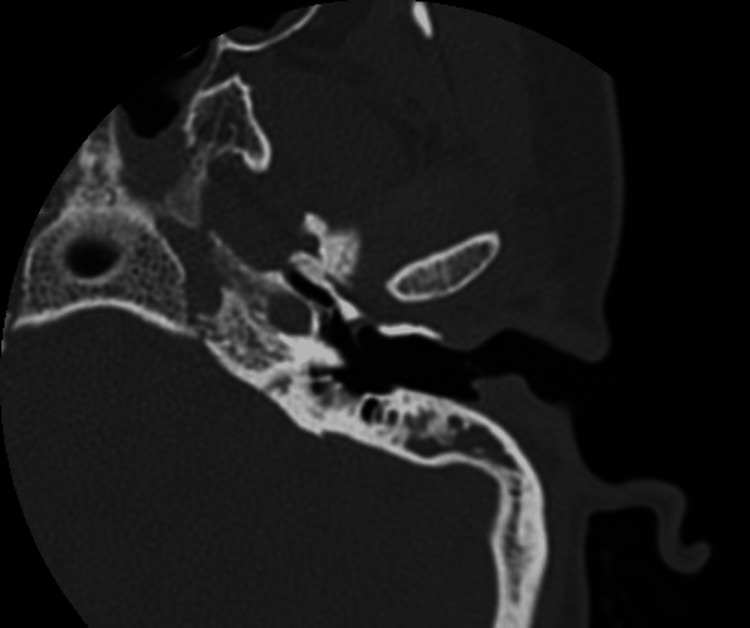
Sample error image of a misclassified SCOM CT scan image using the Neural Network model SCOM: suppurative chronic otitis media; CT: computed tomography.

**Figure 5:**
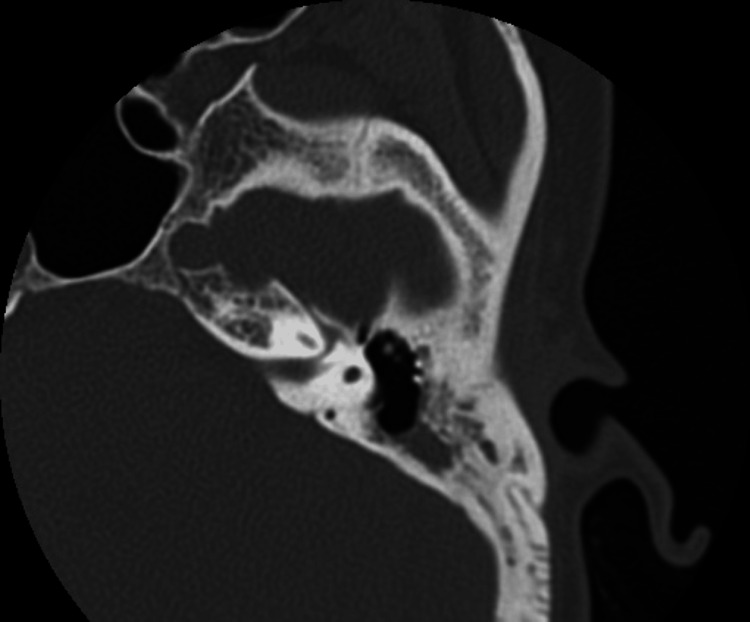
Sample error image of a misclassified SCOM CT scan image using the Logistic Regression model SCOM: suppurative chronic otitis media; CT: computed tomography.

**Figure 6:**
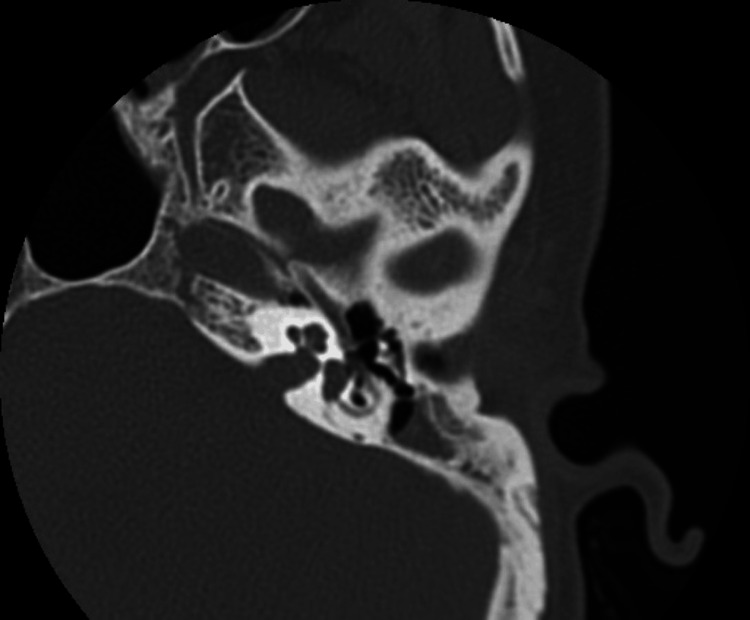
Sample error image of a misclassified SCOM CT scan image using the SVM model SCOM: suppurative chronic otitis media; CT: computed tomography; SVM: Support Vector Machine.

**Figure 7:**
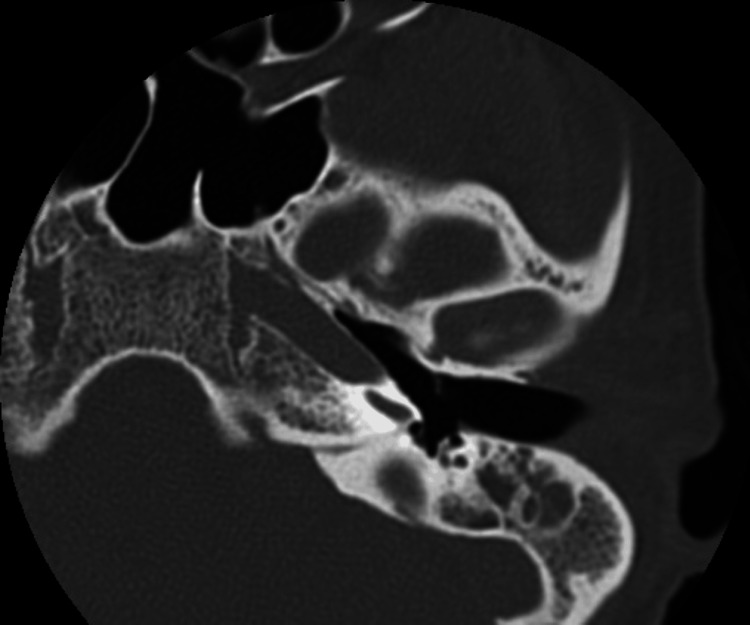
Sample error image of a misclassified SCOM CT scan image using the Random Forest model SCOM: suppurative chronic otitis media; CT: computed tomography.

We conducted model testing on a separate dataset, and the results are summarized in Table 7. Sample error images include temporal bone CT scans where SCOM was mistaken for middle ear cholesteatoma (Figures 3-7). 

**Table 7:** Misclassification counts and sample error images for various supervised machine learning models kNN: k-Nearest Neighbors; ReLu: Rectified Linear Unit; Adam: Adaptive Moment Estimation; Lasso: Least Absolute Shrinkage and Selection Operator.

Model	Misclassified	Total instances	Regularization/parameters	Sample error Images
kNN	45	125	k=5 Euclidean metric	Figure 3
Neural Network	4	125	Activation function: ReLu Solver: Adam	Figure 4
Logistic Regression	5	125	Regularization: Lasso (L1)	Figure 5
SVM	7	125	Not applied	Figure 6
Random Forest	28	125	Not applied	Figure 7

The Neural Network model exhibited the best performance with the lowest number of misclassifications, followed closely by logistic regression with Lasso regularization. Then, the SVM model. These three models appear to be well-suited for this dataset, demonstrating strong generalization capabilities. 

Random Forest model performed moderately well, but the relatively higher number of errors suggests that it may require further tuning or that its underlying assumptions and mechanisms were not as well aligned with the data structure compared to the top-performing models.

On the other hand, while the kNN model showed the highest performance on the training-validation dataset, it performed poorly on the second independent dataset, despite adjustments to the "k" value and changes to the distance metric, including Manhattan, Chebyshev, and Mahalanobis distances instead of the usual Euclidean distance. Although the second dataset shares similar characteristics with the training-validation dataset, the limited performance of kNN on this dataset suggests inherent limitations within the model that prevent it from generalizing effectively. This indicates that kNN may not be the most suitable model for this problem, even with parameter adjustments. 

## Discussion

ML research involves various scientific disciplines, bringing together diverse concepts from computational learning theory, artificial neural networks, statistics, stochastic modeling, genetic algorithms, pattern recognition, and image mining.

In ML, generative and discriminative models represent two distinct approaches to data modeling. Generative models, such as Bayesian classifiers, Hidden Markov Models, Gaussian Mixture Models, and GANs, are designed to model how data is generated and are useful in applications where simulating new data points is essential, such as in drug discovery or image generation [12,14-16]. On the other hand, discriminative models, including Logistic Regression, SVM, most neural networks, and decision trees, focus on making accurate predictions, especially for classification and regression tasks, without attempting to understand the underlying data generation process [12,14-16].

Image mining involves the extraction of hidden information, analysis of image statistics, and identification of patterns not explicitly present in images. This interdisciplinary field combines techniques from computer vision, image processing, data mining, databases, ML, and AI [17,18]. A key feature of image mining is its ability to identify significant patterns without prior knowledge of those patterns. Large image databases are often used for rule mining, and extensive research has been conducted in this area. The mining processes rely on integrated collections of images and their associated data [12,19,20].

In medicine, computer-based medical imaging systems enhance diagnostic assistance by utilizing doctors' expertise to identify diseases using minimally invasive techniques such as CT scans, ultrasonography, endoscopy, confocal microscopy, radiography, and MRI [16,21,22]. Medical image mining algorithms focus on extracting measurable features from large datasets or improving the quality of medical images, such as CT scans, X-rays, or MRIs, which may contain malignant nodules, tumors, or lesions. These algorithms typically operate in three stages to improve diagnostic accuracy [16,21,22].

The initial phase of medical image mining involves generating candidates by identifying pathological ROI within the medical image, which are then labeled as candidates. This process primarily uses image processing algorithms designed to detect distinct variations in the image. Alternatively, it can be done manually, but manual identification is time-consuming and may introduce subjectivity, leading to variability and inconsistency due to differing interpretations or fatigue of the examiner [23,24].

Algorithms used for segmenting the ROI may include thresholding algorithms, edge detection algorithms, deep learning models, clustering algorithms, and more [25,26].

The second phase focuses on feature extraction, where descriptive morphological and texture features, along with embedding vectors, are computed for each candidate using advanced image processing techniques [25-29]. In this study, we utilized the Inception V3 perceptron, pre-trained on the ImageNet Dataset, to extract embedding vectors, which represent key characteristics of the images in numerical form. However, embedding models are often seen as "black boxes" due to their limited transparency in explaining how decisions are made [30].

The final phase of image database classification involves classifying candidates based on their extracted feature vectors, which is crucial to the overall effectiveness of image analysis systems. For this purpose, ML discriminative models such as Logistic Regression, SVM, neural networks, decision trees, and Random Forest are commonly employed [16]. These supervised models rely on a labeled dataset for the training-validation phase, where experts have already identified and labeled different classes, ensuring accurate classification during analysis.

In this study, we applied several supervised discriminative ML models to diagnose middle ear cholesteatoma using temporal bone CT scans, focusing on evaluating model performance through comprehensive metrics and misclassification analysis. A key challenge was data imbalance, with the cholesteatoma group comprising only 16% of the database. Additionally, the dataset included images of both left and right ears, with inconsistent landmarks due to the serial nature of temporal bone CT scans. To address these issues, we mirrored right-side images to the left and applied oversampling for data augmentation. This approach generated high-quality new candidates, but the inherent variability in temporal bone anatomy remained unresolved [16].

Our findings suggest that both kNN and Neural Network models achieved excellent performance in internal cross-validation, with AUC scores of 1.000 and F1 scores above 0.996. However, while the Neural Network maintained high performance during external validation (only four misclassified images out of 125), the kNN model exhibited a significant performance drop, misclassifying 45 of 125 cases (36% error rate). This discrepancy suggests that the kNN model may be prone to overfitting and lacks robustness on unseen data. Thus, within the scope of our dataset and modeling strategy, its clinical applicability appears limited in this specific use case, and caution should be exercised before considering its deployment in real-world settings. In contrast, the Neural Network demonstrated consistent performance and generalizability, supporting its potential reliability for real-world clinical decision-making.

Although Logistic Regression and SVM did not perform as well as kNN and Neural Networks on the training-validation dataset, they still demonstrate strong performance with F1 scores above 0.97. This robustness was further validated by classifying the second dataset, with only five out of 125 and seven out of 125 images misclassified, respectively.

Regarding the logistic regression model, interpretability was addressed through the use of L1 (Lasso) regularization, which eliminates noncontributing variables by setting their coefficients to zero. This yields a sparse and clinically readable model, in which the direction and magnitude of each selected feature’s effect can be directly interpreted.

Given the intrinsic interpretability of logistic regression and the transparent effect of Lasso-based feature selection, post hoc interpretability tools such as SHAP or LIME were not applied, as they were considered redundant in this specific context. Future studies involving more complex or nonlinear models may benefit from their inclusion.

Random Forest models, while generally effective, did not perform at the same level as the former classifier either on the training-validation set or the independent testing dataset. The relatively lower performance of the Random Forest model may be due to the nature of the feature space generated by the Inception V3 embeddings, which is already compact and high-level. Such embeddings may not benefit from the feature-splitting mechanisms of decision trees, leading to reduced ensemble diversity.

Future studies may explore advanced ensemble techniques such as LightGBM, a gradient boosting framework that is computationally efficient and often outperforms traditional random forests on structured or embedded data. Its use in CT image-based classification of otological pathology could provide improved accuracy and speed with appropriate parameter tuning. 

Despite achieving high-accuracy metrics, the kNN model exhibited a considerable number of misclassifications on a secondary dataset. Efforts to improve its performance through adjustments to the distance metrics, including the use of Manhattan, Chebyshev, and Mahalanobis distances, and varying the parameter "k," were unsuccessful. This lack of improvement was observed despite the second dataset sharing similar characteristics with the initial training-validation dataset. This suggests that the kNN algorithm may not be ideally suited for this particular dataset. Challenges potentially stem from kNN's inherent limitations, such as its sensitivity to high-dimensional spaces, dependence on local decision-making, and difficulty in capturing complex data interactions effectively.

One methodological limitation of our study involves the selection of hyperparameters for the ML models. Although we used grid search combined with 10-fold cross-validation, both considered standard practices in clinical ML applications, the process still depends on manually defined search ranges. This introduces a degree of arbitrariness that may influence model performance and reproducibility. Future studies could explore more adaptive hyperparameter optimization techniques, such as Bayesian optimization or Automated Machine Learning (AutoML) frameworks. These approaches dynamically guide the search process based on model performance, potentially improving both tuning efficiency and generalizability across different datasets. Additionally, our study is limited by its single-center data source and manual ROI annotations. Expanding to multi-institutional datasets and integrating automated segmentation techniques may further enhance the robustness and clinical applicability of the proposed models. 

Another limitation of this study is the use of manual segmentation for selecting the ROI. While this approach allowed for precise control and clinically relevant interpretation of partial middle ear filling, it may introduce inter-operator variability and limit scalability. Unlike algorithmic segmentation, manual methods are not easily reproducible across large datasets or automated pipelines. However, in this study, segmentation was performed by experienced clinicians using consistent anatomical criteria, and the use of embedding extraction via a deep learning model (Inception V3) mitigated potential variability by encoding global image features. Therefore, while the manual approach may not be optimal for large-scale deployment, it was appropriate and reliable for our diagnostic modeling objectives.

Additionally, while data imbalance was addressed using image mirroring and oversampling, advanced synthetic augmentation techniques such as GANs were not applied. The development and validation of GANs specifically adapted to temporal bone CT data remain areas for future investigation and may improve data diversity and model generalization.

In the literature, differential diagnosis between SCOM and middle ear cholesteatoma is an ongoing challenge. Several studies have explored this issue based on automated classification of endoscopic or microscopic tympanic images [31-33]. However, only a few studies have addressed this task using standard temporal bone CT scan images [6-11]. The methodologies varied, employing either deep learning models, ML models, or a combination of both, and generally produced satisfactory results. Although CNNs were used in nearly all these studies, SVM was the only additional ML tool explored. Our study suggests that, in addition to neural networks, ML models such as SVM and Logistic Regression are also suitable for identifying middle ear cholesteatoma based on temporal bone CT scan images. Further research using Random Forest and kNN models on a broader range of CT scan images is needed to draw conclusions about the effectiveness of these models in identifying middle ear cholesteatoma.

## Conclusions

To sum up, this study underscores the potential of advanced ML models in medical imaging diagnostics, but also highlights the importance of model selection and parameter tuning. Further research should explore the integration of these models into clinical workflows, the impact of dataset variability, and model generalizability across different populations and imaging technologies.
